# Automated Facial Emotion Recognition System Detects Altered Emotional Processing During Craving Induction in Individuals with Substance Use Disorder

**DOI:** 10.3390/healthcare14101422

**Published:** 2026-05-21

**Authors:** Joaquin García-Estrada, Diana Emilia Martínez-Fernández, Iris del Socorro Pérez-Alcaraz, Carlos Joel Mondragón-Gomar, Irene G. Aguilar-García, Sonia Luquin, David Fernández-Quezada

**Affiliations:** 1 Instituto de Neurociencias Traslacionales, Departamento de Neurociencias, Centro Universitario de Ciencias de la Salud (CUCS), Universidad de Guadalajara (UdeG), Sierra Mojada 950, Guadalajara 44340, Mexico; 2 Instituto Transdisciplinar de Investigación y Servicios (ITRANS), Universidad de Guadalajara (UdeG), Zapopan 45150, Mexico; 3 Centro Integral de Intervención Social, Fundación México Me Necesita A.C., Cajititlán 45670, Mexico

**Keywords:** craving, emotional expressions, substance use disorder, FER, addiction

## Abstract

**Background**: Substance Use Disorder (SUD) is characterized by recurrent craving episodes frequently associated with emotional dysregulation and altered reward processing. This study aimed to evaluate whether emotional states associated with craving episodes can be detected through automated facial emotion recognition during controlled emotional induction. **Methods**: Forty-one participants completed a 14-day ecological momentary assessment (EMA) monitoring anxiety and craving levels, followed by an emotional induction task using standardized stimuli from the EmoMadrid database and addiction-related images. Facial expressions were recorded and analyzed in real time using a computational facial emotion recognition model trained on the FER-2013 dataset. **Results**: Participants with SUD exhibited significantly reduced positive emotional valence and emotional activation in response to positive stimuli compared with healthy controls (HC), with large effect sizes observed for emotional valence (Hedges’ g = 1.76) and emotional activation (Hedges’ g = 1.33). Item-level analyses revealed that most between-group differences occurred in stimuli depicting social interactions. Individuals with SUD also showed higher frequencies of fear-related facial expressions and lower frequencies of disgust-related expressions compared with HC, with moderate effect sizes observed for both emotional dimensions (Hedges’ g = 0.72; *p* = 0.02). **Conclusions**: These results suggest that people with SUD have changes in how they process emotions, showing less response to positive things and unique facial expressions related to craving. However, given the relatively modest and clinically heterogeneous sample, the findings should be interpreted cautiously and require replication in larger and more homogeneous populations.

## 1. Introduction

SUD is a chronic, relapsing neuropsychiatric condition characterized by persistent and compulsive substance use despite significant adverse physical, cognitive, behavioral, and social consequences [1,2]. The disorder is maintained by maladaptive reinforcement processes that progressively induce long-lasting neurofunctional alterations, particularly within circuits implicated in reward processing, stress regulation, and executive control. These neuroadaptations often persist beyond detoxification or rehabilitation, contributing to high relapse rates and sustained vulnerability.

Beyond its clinical burden at the individual level, SUD constitutes a major public health concern worldwide. It is associated with increased mortality, elevated risk of chronic and acute medical conditions, and substantial socioeconomic costs [3]. In Mexico, for instance, SUD-related expenditures and productivity losses represent a significant proportion of national economic resources [4,5].

A central feature of SUD is craving, defined as an intense desire or urge to consume a substance, triggered by recalling prior rewarding experiences and negative emotional states. This phenomenon directly results from the interaction between dysregulated reward circuitry and altered stress- and emotion-related neural systems [6]. Converging evidence demonstrates that individuals with SUD consistently exhibit heightened negative emotionality and impaired emotion regulation capacities compared to healthy controls. Chronic substance exposure has been associated with functional and structural alterations in neural networks encompassing the amygdala, prefrontal cortex, insula, and striatal regions, areas critically involved in emotional processing, interception, and inhibitory control [7]. These alterations may increase vulnerability to mood disturbances and impair adaptive regulatory mechanisms [8,9].

Emotion dysregulation is increasingly recognized as a risk factor and maintaining mechanism in addiction [10,11]. It involves experiencing intense affective states, heightened physiological arousal, and insufficient regulatory capacity. These factors may lead to maladaptive coping strategies such as substance use [12]. Thus, interventions aiming to improve emotion regulation specifically target the underlying processes that sustain addictive behaviors, making impaired emotion regulation a promising focus for therapeutic efforts.

Craving episodes are frequently precipitated by contextual cues, including specific people, environments, objects, or activities previously associated with substance use [13]. These cues can elicit powerful conditioned emotional and motivational responses, even after prolonged periods of abstinence, thereby facilitating relapse [14]. Experimental paradigms that reliably induce emotional reactivity are therefore critical for understanding the mechanisms underlying cue-induced craving. The EmoMadrid database (An Emotional Pictures Database for Affective Research) provides a standardized set of visual stimuli validated in Spanish-speaking populations and systematically categorized along core affective dimensions, including valence, arousal, and dominance. The use of validated visual stimuli allows controlled induction of emotional states with well-characterized psychometric properties, enhancing experimental rigor and reproducibility [15].

Visual exposure was selected in the present study due to the high sensitivity of the human visual system to emotionally noticeable (salient) stimuli. Emotional reactivity to visual cues involves coordinated activation of emotional (affective), memory-related (mnemonic), and behavioral response systems. This activation is influenced both by the inherent features of the stimulus (its intrinsic properties) and how each person interprets the context [16]. Such research setups (paradigms) offer a controlled imitation of real-world cue exposure while maintaining experimental consistency (standardization).

In this study, we investigated whether emotional states linked to craving episodes can be detected through facial emotion recognition. We used an emotional induction task, which involves deliberately eliciting specific emotions using standardized pictures from the EmoMadrid database and images related to addiction. By combining controlled emotional induction, ecological momentary assessment (real-time data collection of participants’ feelings and behaviors in their natural environments), and computational analysis of facial expressions (using computer-based methods to evaluate and quantify expressions), we aimed to identify objective markers of craving-related emotional states in individuals with substance use disorder.

## 2. Materials and Methods

All procedures were approved by the Ethics and Research Committees of the Centro Universitario de Ciencias de la Salud, Universidad de Guadalajara, and conducted in accordance with the Declaration of Helsinki. Prior to testing, subjects were briefed on the study’s aims and required to sign informed consent forms. All participants provided their consent.

### 2.1. Study Design

This study employed a comparative observational design combining longitudinal EMA, a controlled emotional induction task, and automated facial emotion recognition (FER) analysis during 14 consecutive days.

### 2.2. Sample Size

The sample size for this study was derived from our previously published protocol 18, from which this analysis represents a focused component on emotional expression during craving. A priori sample size estimation was performed using Epidat 4.2 for the comparison of two independent means assuming unequal variances, a 1:1 allocation ratio, α = 0.05, and statistical power of 0.80. The calculation was based on expected between-group differences and variability parameters reported in the reference study by Rykov et al. The estimated minimum sample size was 17 participants per group. To account for potential attrition during EMA monitoring and clinical follow-up, 22 participants were initially recruited per group.

### 2.3. Population

Participants were recruited from specialized addiction treatment facilities. The clinical sample consisted of individuals diagnosed with SUD who were actively undergoing rehabilitation treatment at Fundación México Me Necesita. A matched group of healthy controls (HC) was recruited for comparison. At enrollment, all participants were assigned a unique identification code to ensure anonymity throughout the study and to ensure secure data management. A total of 44 participants were initially assessed at intake, with 22 individuals (50%) allocated to the SUD group and 22 (50%) to the HC group. During the study, 2 participants in the HC group (10%) withdrew due to the burden of daily ecological momentary assessment (EMA) self-reports. Additionally, one participant from the SUD group (5%) was excluded because of severe extrapyramidal side effects related to antipsychotic medication, primarily dystonia and Parkinsonism, which interfered with the accurate use of the Facial Emotion Recognition (FER) software Version 1.0. After attrition, 41 participants completed the full study protocol. All remaining participants (100%) completed the midpoint emotional assessment, and 40 participants (95.24%) completed the daily EMAs across the study period. The final analyzed sample comprised 41 participants aged 25–31 years. Inclusion criteria for the SUD group consisted of a confirmed clinical diagnosis of SUD and active participation in a rehabilitation program at the time of recruitment. For all participants, additional inclusion criteria included the ability to provide written informed consent and the absence of physical limitations that could interfere with the use of the smartwatch or the automated FER system. Exclusion criteria included facial paralysis, craniofacial malformations (e.g., cleft lip or palate), prior facial surgeries affecting muscle function, or severe motor disturbances that could compromise accurate facial emotion recording.

### 2.4. Emotional Arousal Image Bank

Emotional induction used stimuli from the EmoMadrid database 15. This database was developed by the Cerebro, Afecto y Cognición (CEACO) research group at the Universidad Autónoma de Madrid. EmoMadrid is a standardized, validated collection of 1200 images for psychological and neuroscientific research. The images fall into three affective categories: positive (*n* = 400), neutral (*n* = 400), and negative (*n* = 400). Each image is normatively rated for valence, arousal, and dominance, which allows for controlled induction of emotional responses. From the full database, 43 images were selected for presentation to participants, ensuring representation across the three affective categories (positive, negative, and neutral) while maintaining the normative thresholds established in the EmoMadrid validation protocol. Selection criteria were based on standardized valence and arousal ratings to ensure clear affective differentiation between categories. In addition to the standardized stimuli, seven substance-related images depicting methamphetamine, alcohol, cocaine, prescription drugs, and tobacco use were incorporated to increase ecological validity and simulate addiction-relevant contextual cues. These additional images were selected to maintain visual quality and presentation standards consistent with the EmoMadrid protocol. Each image was presented for 1 s, followed by a brief interstimulus interval during which participants evaluated perceived emotional valence (type of emotion induced) and emotional intensity (arousal). To minimize visual and emotional fatigue, 30-s rest periods were provided after every 25 images. The entire session was recorded in .mp4 audiovisual format. Facial expressions were continuously monitored and quantified in real time using a custom-developed computational script designed for automated Facial Emotion Recognition Software. This procedure enabled the simultaneous capture of subjective emotional ratings and objective facial expression data during controlled emotional induction [17].

### 2.5. Modified Mannheim Craving Scale

Craving intensity was assessed using a modified version of the Mannheim Craving Scale [18]. This self-report instrument is designed to evaluate cognitive and affective components associated with substance use following periods of abstinence. The scale consists of 16 items that assess intrusive thoughts, urges to consume substances, emotional distress related to abstinence, and perceived control over substance-related impulses. It evaluates dimensions such as frequency and intensity of craving episodes, anxiety or irritability associated with the urge to consume, and the individual’s perceived capacity to resist or regulate the impulse. For the purposes of the present study, a partial administration of the instrument was implemented during the emotional assessment session. For the purposes of the present study, a targeted administration of selected Mannheim Craving Scale items was implemented during the emotional assessment session. Specifically, items 9 through 12 were selected because they assess craving intensity, emotional discomfort associated with craving, and perceived impulse control, dimensions considered directly relevant to the emotional induction paradigm. This partial administration was intended to reduce participant burden and minimize fatigue during repeated emotional assessments in a clinical rehabilitation population. Following exposure to emotionally salient visual stimuli from the validated image database, the same items were re-administered to assess potential changes in craving intensity and impulse control. Two supplementary items were also included: one to determine whether participants perceived any change in emotional state after stimulus exposure, and another to elicit a brief qualitative description of that change. This approach allowed assessment of both quantitative variation in craving-related dimensions and subjective emotional shifts induced by the experimental manipulation.

### 2.6. Facial Expression Recognition System

**Figure 1:**
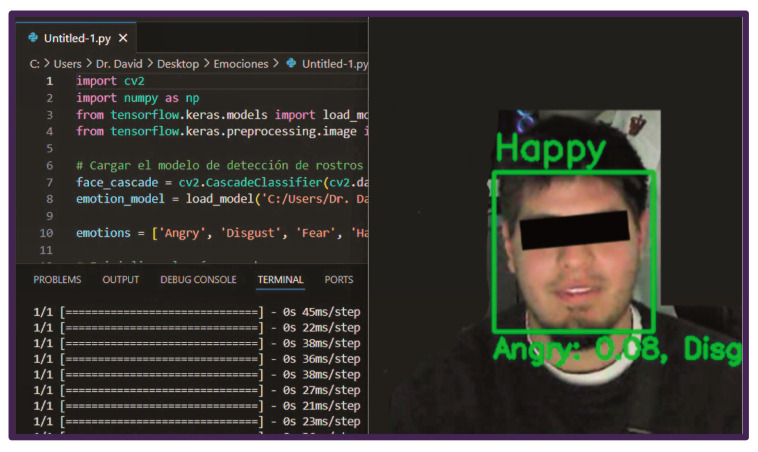
Implementation of the automated facial emotion recognition system. (**Left panel**): Example of the Python script used for facial emotion detection, including the use of the OpenCV library for face detection and a convolutional neural network model trained on the FER-2013 dataset for emotion classification. (**Right panel**): Example output of the system during analysis. The algorithm detects the facial region of interest (green bounding box) and classifies the dominant emotional expression in real time, displaying the predicted emotion label (“Happy”). The participant’s eyes are anonymized to preserve identity. Written informed consent was obtained from the participant for the publication of this image.

The smart system of emotion quantification was implemented to automatically detect and classify participants’ facial expressions during the emotional induction task, as shown in Figure 1. The protocol ran on a desktop equipped with an Intel i7-12650H CPU (2.30 GHz; MSI Cyborg 15 A12V), 64 GB RAM, and an NVIDIA RTX 4050 GPU, enabling real-time processing. The video was acquired with a 1080p webcam at a standardized distance to optimize facial landmark detection and reduce variability from camera angle or lighting. Facial activity was continuously recorded during stimulus presentation. Automatic emotion recognition was performed using a personalized script algorithm implemented in Python 3.10 (Python Software Foundation, Wilmington, DE, USA). Face detection and preprocessing were conducted using OpenCV 4.8.0 (Open Source Vision Library, Intel Corporation, Santa Clara, CA, USA), which extracted facial regions of interest (ROIs) from each video frame. Once detected, faces were converted to grayscale, resized to 48 × 48 pixels, and normalized to match the input specifications of the classification model. Subsequently, emotion classification was performed using a convolutional neural network (CNN)-based facial emotion recognition model trained on the publicly available FER-2013 dataset. FER-2013 is one of the most widely used benchmark datasets in affective computing research due to its standardized emotional labeling and suitability for deep learning applications [19,20]. The dataset contains 35,887 grayscale facial images with a resolution of 48 × 48 pixels categorized into seven emotional classes: anger, disgust, fear, happiness, sadness, surprise, and neutral. The dataset includes substantial variability in facial pose, illumination conditions, and facial characteristics, although it also presents intrinsic limitations such as low image resolution, class imbalance, label noise, and the predominance of posed facial expressions under controlled acquisition conditions [19,21]. FER-2013 has been extensively used for the development and evaluation of convolutional neural network (CNN)-based emotion recognition systems because of its suitability for automated feature extraction and real-time computational implementation. Detected facial regions were extracted using OpenCV-based face detection algorithms and subsequently converted to grayscale, normalized, and resized to 48 × 48 pixels to match the FER-2013 input format and reduce variability associated with illumination and image scaling. Emotion probabilities were computed on a frame-by-frame basis, enabling continuous monitoring of affective responses during stimulus exposure. For each participant, the system generated real-time outputs indicating the dominant detected emotion and cumulative counts for each emotional category across the experimental session. All scripts developed for facial detection and emotion classification are available in Supplementary Materials S1.

### 2.7. Statistical Analysis

Statistical analyses were performed in R (R Foundation for Statistical Computing, Vienna, Austria). Normality of distributions was assessed using the Shapiro–Wilk test. Between-group comparisons for continuous variables were conducted using independent-samples t-tests or Mann–Whitney U tests when normality assumptions were not met. Categorical variables were compared using chi-square tests. Longitudinal EMA data were analyzed using repeated measures comparisons of daily mean values between groups. Linear regression analyses were performed to evaluate trends across the 14-day period. Statistical significance was set at *p* < 0.05.

## 3. Results

### 3.1. Characteristics and Adherence of Participants

Out of the 44 participants enrolled in intake, 22 (50%) belonged to the SUD group and 22 (50%) to the HC group. During the study, 2 participants from the HC group (10%) withdrew due to the burden associated with completing daily EMA self-reports. Additionally, 1 participant from the SUD group (5%) was excluded because severe extrapyramidal side effects of antipsychotic medication, primarily dystonia and parkinsonism, interfered with the use of the Facial Emotion Recognition software. Of the remaining participants, 41 individuals completed the midpoint emotional assessment, and 40 participants (95.24%) completed the daily EMAs throughout the duration of the study, as shown in Table 1.

Participants in the SUD group ranged in age from 18 to 55 years (mean = 31.33, SD = 10.01), whereas those in the HC group ranged from 19 to 38 years (mean = 25.7, SD = 6.39). Educational attainment differed between groups. In the SUD group, the highest level of education ranged from secondary school (14.29%) to postgraduate studies (9.52%), with the largest proportion reporting high school completion (47.62%), followed by undergraduate education (23.81%). In contrast, most HC participants had completed a bachelor’s degree (70%), followed by postgraduate education (25%) and high school education (1%; Table 1).

Marital status was predominantly single in both groups (SUD: 76.19%; HC: 85%). However, notable differences were observed between groups: 19.05% of participants in the SUD group reported being separated or divorced, whereas only 4.76% reported currently being in a relationship. In the HC group, 15% reported being in a long-term relationship, and none reported a prior separation or divorce (Table 1). Regarding living arrangements prior to entering the rehabilitation program, most participants in the SUD group reported living with their parents and siblings (47.62%), followed by living alone (19.05%), living with other relatives (19.05%), living with a partner and children (9.52%), and living with roommates (4.76%). In the HC group, the majority also reported living with their parents and siblings (60%), followed by living with partners and children or with unrelated roommates (15% each), and a smaller proportion living alone or with other relatives (5% each) (Table 1).

Both groups presented several neuropsychiatric conditions, with depression being the most prevalent (SUD = 52.38%, HC = 10%), followed by anxiety disorders (SUD = 42.86%, HC = 20%) and attention-deficit/hyperactivity disorder (ADHD) (SUD = 28.57%, HC = 15%). Additional conditions observed exclusively in the SUD group included bipolar disorder (19.05%) and one case of antisocial personality disorder (4.76%), as shown in Table 1.

Regarding pharmacological treatment, five participants in the SUD group (23.81%) reported not using medication. Of the 17 remaining participants, medications included antipsychotics (42.86%), antidepressants (14.29%), anxiolytics (2%), methylphenidate (9.52%), and antiretroviral therapy (4.76%), administered in various combinations. In the HC group, three participants (15%) received pharmacological treatment: methylphenidate (10%) or anxiolytics (5%) (see Table 1).

### 3.2. Substance Use Characteristics in the SUD Group

Participants in the SUD group were predominantly polysubstance users. Most used methamphetamine (*n* = 18, 85.71%), followed by alcohol (*n* = 2, 9.52%) and cocaine (*n* = 1, 4.76%). Mean duration for primary substance use was 7.85 years (SD = 5.93) for methamphetamine, 12.50 years (SD = 16.26) for alcohol, and 5 years for cocaine.

Participants also reported the use of multiple secondary substances. Frequently co-used substances were alcohol (76.19%), tobacco and inhalants (66.67%), cannabis (61.90%), and cocaine (57.14%). Less frequently reported substances formed a separate group: hallucinogens, sedatives, and unclassified stimulants (23.81%).

The age of onset of substance use ranged from 6 to 48 years, with a mean onset age of 14.81 years (SD = 8.39). The most frequently reported age of initiation was 15 years (*n* = 4). The most common gateway substances were alcohol (52.38%), tobacco (47.62%), and cannabis (4.76%), as shown in Supplementary Materials S2.

### 3.3. Anxiety Influences Craving Levels in SUD

**Figure 2:**
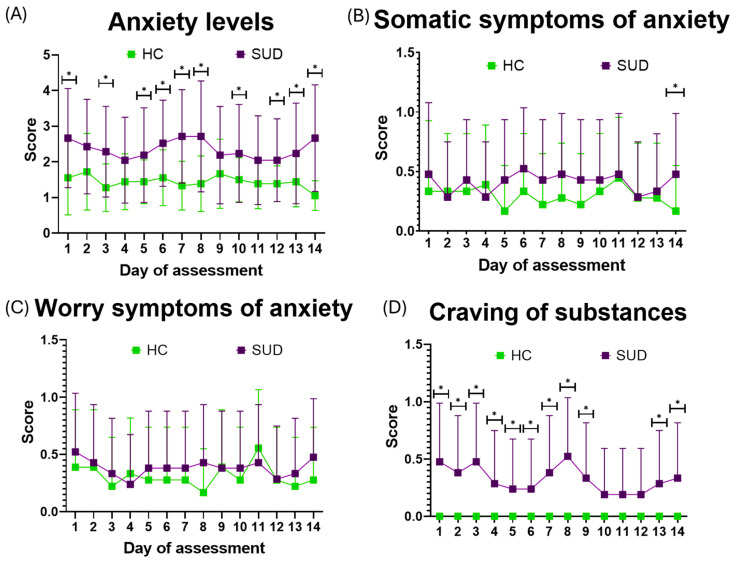
Daily EMA measures anxiety and craving in HC and SUD groups across the 14-day observation period. Panels show mean ± SD values for (**A**) anxiety levels, (**B**) somatic anxiety symptoms, (**C**) worry symptoms, and (**D**) craving. Somatic and worry symptoms were coded as presence (1) or absence (0). Green markers represent the HC group, and purple markers represent the SUD group. Asterisks indicate statistically significant between-group differences for the corresponding day (* *p* < 0.05).

Across the 14-day EMA period, craving levels differed significantly between HC and SUD groups on 11 days, and anxiety scores on 10. In contrast, significant somatic anxiety differences appeared only on Day 14 (*p* = 0.04); on other days, somatic symptoms were comparable. No significant differences emerged for worry at any point. Craving and general anxiety showed the most consistent group differences, while somatic symptoms and worry remained largely comparable (Table 2, Figure 2).

**Figure 3:**
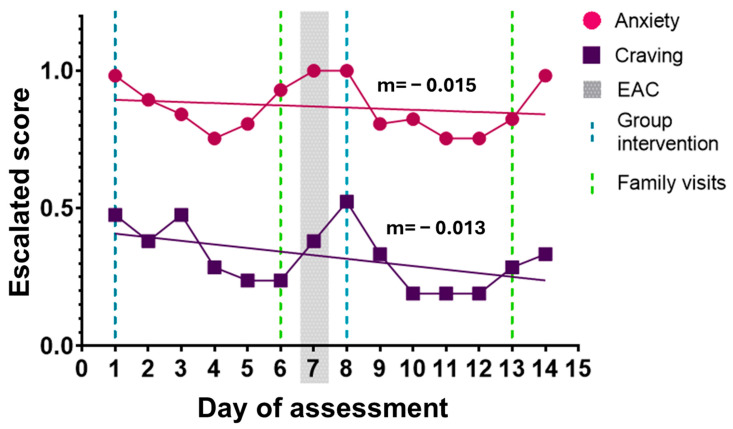
Trends in anxiety and craving among participants in the SUD group across the monitoring period. Daily escalated scores for anxiety (circles) and craving (squares) are shown across the observation period. Solid lines represent linear trend lines for anxiety (m = −0.015) and craving (m = −0.013). Blue dashed vertical lines indicate days of group therapy interventions (Days 1 and 8), green dashed vertical lines indicate family visitation days (Days 6 and 13), and the shaded gray area represents the Emotional Activation and Craving (EAC) test conducted on Day 7.

Simultaneously, higher anxiety levels in the SUD group appeared to coincide with specific scheduled events during the monitoring period, including family visitations on Sundays (Day 6: 2.52 ± 1.21; Day 13: 2.24 ± 2.42), therapeutic sessions involving family members (Days 1 and 8), and the emotional activation craving test (Day 7). Increased craving intensity was also observed on the day following several of these events, although this pattern was descriptive in nature. Across the observation period, a slight decreasing trend was observed for anxiety (slope = −0.015, *p* = 0.66) and craving (slope = −0.013, *p* = 0.57), although neither trend reached statistical significance, (Figure 3).

### 3.4. Positive Emotional Valence and Activation Are Diminished in SUD

Separate analyses of emotional valence and activation were conducted within each group for the four types of visual stimuli, and the results were then compared between groups. A significant difference in the valence ratings of positive stimuli (Δ = −0.63, *p* < 0.01) indicated that HC participants rated these stimuli as more positive ($\bar{x}$ = 3.90, SD = 0.26), while SUD group participants rated them as less positive ($\bar{x}$ = 3.27, SD = 0.42). This difference was associated with a large effect size (Hedges’ g = 1.76), suggesting a substantial reduction in positive emotional responsiveness in individuals with SUD. No significant between-group differences were observed for negative (Δ = 0.03, *p* = 0.75), neutral (Δ = −0.12, *p* = 0.30), or substance-related stimuli (Δ = 0.03, *p* = 0.78).

**Figure 4:**
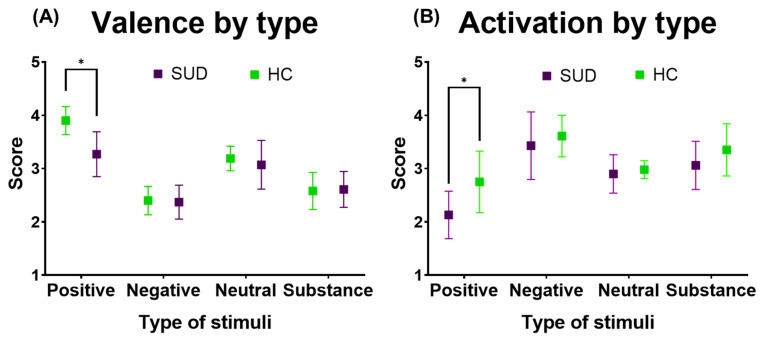
Emotional valence and activation ratings by stimulus type in SUD and HC groups. (**A**) Mean ± SD emotional valence scores for the four stimulus categories (positive, negative, neutral, and substance-related). (**B**) Mean ± SD emotional activation scores for the same stimulus categories. Asterisks indicate statistically significant between-groups for the corresponding day (* *p* < 0.05). Upon analysis by item, it was possible to determine the specific items where differences were found, as shown in Supplementary Materials S3.

Emotional activation showed a similar pattern when analyzed by stimulus type. Positive stimuli were rated as significantly less activated by participants in the SUD group ($\bar{x}$ = 2.10) compared with HC participants ($\bar{x}$ = 2.80; Δ = −0.62, *p* < 0.01). This difference also demonstrated a large effect size (Hedges’ g = 1.33). No significant between-group differences were observed for negative (Δ = 0.18, *p* = 0.29), neutral (Δ = 0.08, *p* = 0.38), or substance-related stimuli (Δ = 0.29, *p* = 0.06) (Figure 4).

**Figure 5:**
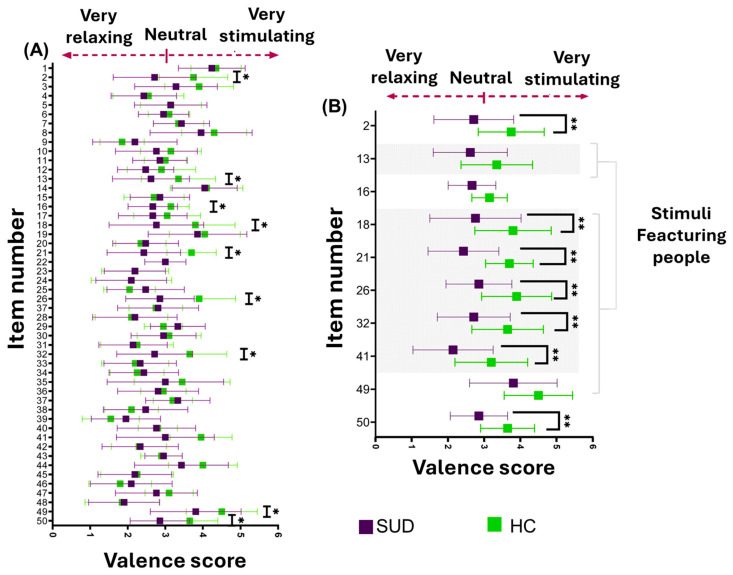
Item-level comparison of emotional valence ratings between SUD and HC groups. (**A**) Mean ± SD valence scores for each stimulus item. Scores ranged from 1 (very negative) to 5 (very positive), with 3 representing neutral valence. Purple markers indicate the SUD group, and green markers indicate the HC group. Asterisks (*) denote items with significant between-group differences. (**B**) Subset of items showing significant differences between groups. Shaded rows indicate stimuli belonging to the “people” category in the image index. Double asterisks (**) indicate items with stronger statistical significance (*p* < 0.01).

Of the ten items showing significant group differences, 60% were in the “people” subcategory, 20% in “food,” and 10% each in “objects” and “animals.” In the “people” category, SUD participants rated all scenes less positively than HC participants (Figure 5). The greatest differences occurred with item 21 (physical intimacy), followed by items 26 (Δ = −1.0) and 18 (Δ = −1.0), both of which depict groups involved in recreational activities (Figure 5).

Significant differences between groups were observed in emotional activation for positive stimuli (Δ = −0.62, *p* < 0.01), with participants in the SUD group reporting lower activation ($\bar{x}$ = 2.10, SD = 0.45) compared with the HC group ($\bar{x}$ = 2.80, SD = 0.58), as shown in Supplementary Materials S3.

**Figure 6:**
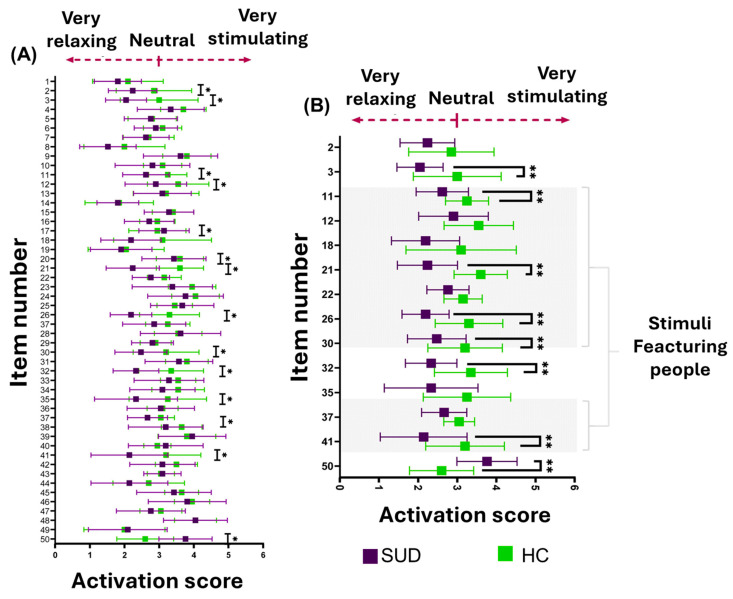
Item-level comparison of emotional activation ratings between SUD and HC groups. (**A**) Mean ± SD activation scores for each stimulus item. Scores ranged from 1 (low activation or relaxing) to 5 (high activation or stimulating), with 3 indicating neutral activation. Purple markers represent the SUD group, and green markers represent the HC group. Asterisks (*) denote items with significant between-group differences. (**B**) Subset of items showing significant differences between groups. Shaded rows indicate stimuli belonging to the “people” category in the image index. Double asterisks (**) indicate stronger statistical significance (*p* < 0.01).

At the item level, 14 stimuli showed significant between-group differences in emotional activation. Of these, 7 (50%) belonged to the “people” category, 3 (21.43%) to the “objects” category, 2 (14.29%) to “food,” and 1 item each to the “substance use” and “animal” categories (7.14% each) (Figure 6).

### 3.5. Comparison of Facial Emotion Expression Between SUD and HC Groups

The six basic emotions and a neutral state detected by the facial emotion recognition algorithm were recorded and analyzed. No significant difference was observed in total emotional expressions between participants with SUD ($\bar{x}$ = 129.62 ± 14.65) and those in the HC reference group ($\bar{x}$ = 131.60 ± 10.96) (Δ = 3.0, *p* = 0.47).

**Figure 7:**
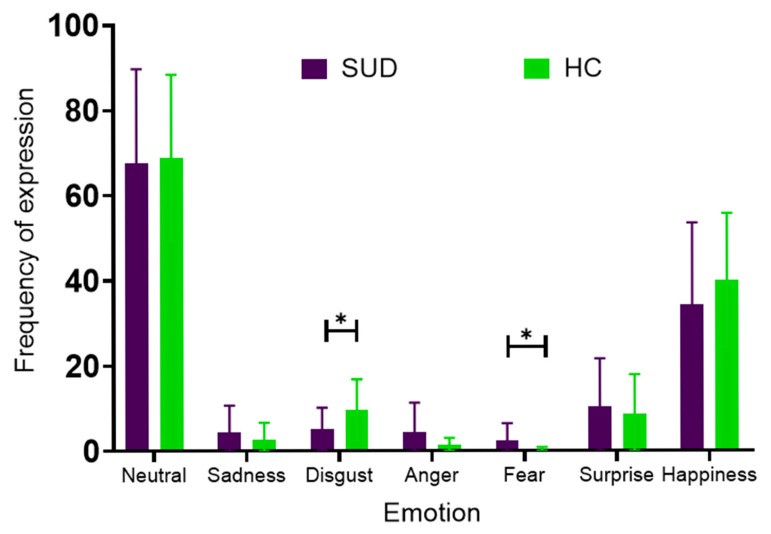
Frequency of facial emotional expressions detected by the smart system in SUD and HC groups. Bars represent mean ± SD counts of emotional expressions detected across the assessment period, from the initial interview (MACS evaluation) to the closing interview. Purple bars correspond to the SUD group and green bars to the HC group. Significant between-group differences were observed for disgust and fear expressions (*p* = 0.02 for both), indicated by asterisks (*).

For individual emotions, SUD participants showed lower rates of disgust ($\bar{x}$ = 5.19 ± 5.01) than HC ($\bar{x}$ = 9.70 ± 7.17; Δ = −4.5, *p* = 0.02), but higher fear ($\bar{x}$ = 2.40 ± 4.06) than HC ($\bar{x}$ = 0.25 ± 0.64; Δ = 2.2, *p* = 0.02), also demonstrating a moderate effect size (Hedges’ g = 0.72). No significant differences were detected for neutral (Δ = −1.3, *p* = 0.84), sadness (Δ = 1.8, *p* = 0.29), anger (Δ = 3.1, *p* = 0.06), surprise (Δ = 3.2, *p* = 0.61), and happiness (Δ = −5.9, *p* = 0.30) (Figure 7).

## 4. Discussion

This study investigated whether emotional states associated with craving episodes in people with SUD can be detected using automated facial emotion recognition. The method used was controlled emotional induction and ecological momentary assessment. By integrating real-time facial emotion recognition, standardized affective stimulation from the EmoMadrid database [15], and ongoing monitoring of anxiety and craving, this study offers new insights into how emotional dysregulation and craving interact in addiction. The results show that people with SUD exhibit lower positive emotional valence and activation when presented with positive stimuli. Their facial expressions reveal more fear and less disgust. These findings suggest that abnormalities in emotional processing are a measurable behavioral trait linked to vulnerability to cravings in SUD.

One of the most consistent findings of the present study was the attenuation of positive emotional responses in participants with SUD. Individuals in the SUD group rated positive stimuli as significantly less positive and less activating. This pattern is consistent with the concept of reward system dysregulation that characterizes chronic addiction [22,23]. Long-term exposure to psychoactive substances induces neuroadaptations within mesocorticolimbic circuits, particularly within dopaminergic projections connecting the ventral tegmental area, nucleus accumbens, and prefrontal cortex [24]. These neuroadaptations reduce sensitivity to natural rewards while increasing motivational salience toward substance-related cues. Therefore, individuals with SUD frequently exhibit blunted hedonic responses to normally rewarding experiences, a phenomenon often referred to as reward deficiency or anhedonia. The reduced positive valence and activation observed in this study likely reflect this broader neurobiological imbalance between reward and stress systems [25].

The SUD group showed notably weaker positive responses to stimuli depicting social interactions and human activities, especially those categorized as ‘people.’ These scenes (recreation, social engagement, and interpersonal intimacy) elicited the most marked dampening of positive response. This pattern highlights a key finding: social reward processing, which depends on neural circuits also implicated in addiction (ventromedial prefrontal cortex, amygdala, striatum) [26,27], is impaired in SUD. Chronic substance use has been linked to reduced responsivity in these circuits to social stimuli, potentially fostering social withdrawal, impaired relationships, and less engagement in non-drug rewards [28]. Thus, diminished emotional responsiveness to socially relevant stimuli may be a behavioral marker of reward system dysfunction in addiction. In contrast to the differences observed for positive stimuli, emotional responses to negative, neutral, and substance-related images did not significantly differ between groups. This pattern suggests that the primary emotional alteration in this cohort may not involve exaggerated reactivity to negative stimuli but rather a diminished capacity to experience positive affect. Such an interpretation aligns with theoretical models proposing that addiction is characterized not only by enhanced negative emotionality but also by reduced sensitivity to natural rewards [29,30]. Within the context of recovery, this imbalance may contribute to persistent motivational deficits and difficulty engaging in adaptive, non-drug-related behaviors.

Another key finding concerns the relationship between emotional dysregulation and craving dynamics captured through EMA monitoring. Across the 14-day observation period, individuals with SUD consistently exhibited higher levels of anxiety and craving compared with healthy controls. These results reinforce the well-established association between negative affective states and substance craving [31,32,33,34]. Emotional distress, particularly anxiety, has long been recognized as a powerful trigger for craving episodes and relapse [35,36,37]. Importantly, in the present study emotional responses were experimentally induced using standardized affective stimuli, allowing controlled examination of emotional reactivity in relation to craving states. This approach enabled integrating subjective emotional ratings with objective behavioral indicators obtained from the automated facial emotion recognition smart system. The use of this computational tool provided an additional layer of behavioral measurement, allowing real-time detection of emotional expressions during the induction task and supporting the validity of the experimental paradigm for studying craving-related emotional processes.

An additional conceptual framework relevant to the present findings is alexithymia, a construct characterized by difficulties in identifying, interpreting, and verbally describing emotional states. Alexithymia has been consistently associated with substance use disorders and emotional dysregulation, particularly in populations with chronic substance exposure and high psychiatric comorbidity [38,39]. Individuals with alexithymia traits frequently exhibit impairments in emotional awareness, interoceptive processing, and affective self-regulation, factors that may directly influence both subjective emotional ratings and observable facial emotional expression patterns [40]. Within the context of the present study, alexithymia may represent an important factor contributing to the reduced positive emotional responsiveness and altered fear- and disgust-related facial expression patterns observed in the SUD group. Difficulties in recognizing and describing internal emotional states could partially affect how participants subjectively evaluated emotional stimuli and how emotional reactions were externally expressed during the emotional induction paradigm.

In addition to subjective emotional responses, the automated facial emotion recognition system revealed alterations in objective facial expression patterns. Although the overall number of emotional expressions did not differ between groups, participants with SUD displayed significantly higher frequencies of fear expressions and lower frequencies of disgust expressions compared with healthy controls. These differences may reflect alterations in emotional processing circuits involved in threat detection and aversive learning. Fear-related responses are strongly associated with amygdala activation and heightened stress sensitivity, both of which are commonly dysregulated in addiction. Increased fear expression may therefore reflect heightened emotional reactivity to internal or external cues associated with craving or withdrawal states [41,42].

Reduced expression of disgust in individuals with SUD is noteworthy. Disgust typically prompts avoidance and self-protection from harmful cues. In addition, diminished disgust toward substance cues may indicate altered judgment of aversive stimuli or impaired inhibition [43,44]. Neuroimaging shows reduced insular activation during disgust in those with dependence, suggesting that altered interoceptive awareness weakens aversive signaling [45]. These findings support the idea that changes in disgust processing may increase addiction vulnerability. However, methodological aspects related to automated facial emotion recognition should also be considered when interpreting these findings. Emotion recognition algorithms more accurately identify emotions with clear facial features, especially when there are distinctive changes in the periocular region. In contrast, several negative emotions share overlapping facial cues, increasing the risk of misclassification. For example, anger may be classified as fear, and disgust as sadness [46]. Thus, while differences in fear and disgust expression could indicate true changes in emotional processing, they may also result from current limitations in FER models.

An additional strength of the present study lies in the use of automated facial emotion recognition technology to quantify emotional responses in real time. Traditional assessments of emotional processing often rely exclusively on self-report measures, which are subject to bias and limited introspective awareness [47]. The integration of computational emotion recognition algorithms allows for objective behavioral measurements that complement subjective reports. The use of machine learning-based facial emotion recognition models trained on large, annotated datasets, such as FER-2013, enables the continuous monitoring of emotional responses with high temporal resolution. Such systems have the potential to transform behavioral assessment in clinical research by providing scalable and objective tools for monitoring emotional states [48,49].

These specific alterations in fear and disgust expressions align with and extend a growing body of literature on emotion recognition deficits in addiction. A pivotal meta-analysis by Castellano et al. [50] synthesized evidence across multiple studies and confirmed that individuals with alcohol and substance use disorders exhibit a significant, though moderate, overall impairment in facial emotion recognition accuracy compared to healthy controls. While that meta-analysis identified a global deficit, the present study’s computational approach allows for a more granular analysis, revealing that this impairment may manifest not as a uniform difficulty but as specific biases in emotional expression, namely heightened fear and blunted disgust. Our findings of reduced disgust expression are particularly resonant with the meta-analysis’s call for research into specific emotion categories, as disgust is crucial for avoidance learning and may be a key but understudied emotion in the context of addiction. The increased fear expression observed in our SUD group further supports the notion of heightened threat sensitivity and altered stress circuitry, providing a behavioral correlate to the neurobiological dysregulation discussed earlier [48].

From a translational perspective, automated emotional monitoring systems may offer valuable tools for addiction treatment and relapse prevention. Individuals with substance use disorders often exhibit impairments in emotional processing and facial emotion recognition, which are associated with altered neural circuits involved in reward processing and emotional regulation [50]. These alterations may contribute to difficulties in identifying emotional states that precede substance-seeking behavior.

Craving episodes frequently emerge rapidly and may be preceded by subtle emotional changes that individuals may not consciously recognize. In this context, automated facial emotion recognition systems could enable real-time detection of emotional patterns associated with increased relapse risk [51]. In rehabilitation, automated emotional monitoring systems could also enhance emotional self-awareness and introspection during treatment by identifying craving-related or distress-associated emotional states in real time. Early detection of high emotional craving states may additionally support the development of adaptive therapeutic interventions aimed at reducing emotional dysregulation and preventing escalation toward substance use. This approach may strengthen patient–clinician communication, support individualized therapeutic decision-making, and contribute to relapse prevention and long-term recovery outcomes.

Importantly, the observed reductions in positive emotional valence and emotional activation were associated with large effect sizes, suggesting substantial differences in reward-related emotional processing between groups. Similarly, moderate effect sizes were observed for fear- and disgust-related facial expressions. However, these findings should be interpreted cautiously due to the relatively modest sample size and the clinical heterogeneity of the SUD population, including differences in psychiatric comorbidities, medication use, and polysubstance exposure. Small samples may increase variability and potentially overestimate effect magnitudes, thereby limiting the generalizability of the findings. Consequently, although the present results provide preliminary evidence supporting altered emotional processing in SUD, larger and more clinically stratified studies are necessary to confirm the reproducibility and representativeness of these observations. Despite these promising implications, several limitations should be considered when interpreting the findings.

First, the facial emotion recognition system used in this study mainly detected emotions from frontal facial images during the craving induction task. Although participants were positioned to maximize frontal face detection, this constraint may limit the system’s performance in natural environments where head movements, occlusions, and non-frontal facial orientations often occur. Real-world emotional interactions involve dynamic and multi-angle facial expressions, and systems trained under controlled laboratory conditions may not fully generalize to these settings. Consequently, the trained model may exhibit reduced sensitivity when detecting spontaneous, subtle, or context-dependent emotional expressions commonly observed in clinical populations. Another methodological limitation concerns the inclusion of independently selected substance-related images that were not part of the validated EmoMadrid database. Although these stimuli were incorporated to increase ecological validity and provide addiction-relevant contextual cues during emotional induction, formal psychometric validation regarding valence, arousal, and dominance dimensions was not performed. Thus, the emotional properties of these images cannot be assumed to be equivalent to those of the standardized EmoMadrid stimuli, which may have introduced variability in the emotional induction paradigm. Additionally, the relatively modest sample size and the clinical heterogeneity of the SUD group, including differences in psychiatric comorbidities, medication use, and polysubstance exposure, may have increased interindividual variability and limited the generalizability of the findings. However, such heterogeneity is highly common in real-world rehabilitation populations, particularly in contexts characterized by polysubstance use and complex psychiatric profiles. The findings should be interpreted cautiously and considered preliminary. Another concern is the partial administration of the Mannheim Craving Scale. Only selected items directly related to craving intensity and emotional control were administered to reduce participant burden during the emotional induction procedure. Consequently, the resulting scores should be interpreted as exploratory indicators of craving-related dimensions.

Second, the FER model in this study was mainly trained on static facial image datasets. While these datasets are common in affective computing research, they do not capture the temporal dynamics of natural emotional expression. Human emotions unfold over time through subtle micro-expressions and dynamic muscle movements. Research has shown that dynamic emotional stimuli can improve emotion recognition compared to static images. This finding indicates that temporal information is important for accurate emotional decoding [52]. Relying on static training datasets may reduce the sensitivity of automated systems. These systems may miss transient or nuanced emotional responses during craving episodes. Additionally, the relatively brief stimulus presentation time may have limited the detection of sustained or delayed emotional responses, particularly in individuals with altered emotional processing.

Third, facial expressions are only one part of emotional communication. Emotional states are conveyed through multiple channels, including body posture, gestures, gaze direction, and environmental context. Studies on substance users have shown that impairments can extend beyond facial expressions to include body-based cues and contextual emotion processing [51]. Without these additional modalities, interpreting facial signals alone may be ambiguous, especially for emotions with overlapping facial features. Integrating information such as body position, head orientation, physiological signals, and scene analysis may greatly improve the accuracy and ecological validity of automated emotion recognition systems. Given the momentary assessment to capture craving-related emotional states, the relatively modest sample size, and the controlled experimental setting, the generalizability of the findings to broader clinical populations may be limited. Previous studies in SUD populations have emphasized the heterogeneity of emotional processing deficits and their relationship with clinical variables such as substance type, duration of use, and social functioning [48,53].

Fourth, methodological consideration concerns the potential demographic and cultural bias associated with the FER-2013 dataset used to train the facial emotion recognition model. Although FER-2013 has been extensively used as a benchmark dataset in affective computing and automated facial emotion recognition research [20], recent studies have highlighted that commonly used FER datasets may present demographic imbalances and variable performance across ethnic and cultural populations [19,54,55]. In particular, unequal demographic representation may influence classification accuracy and emotional recognition stability when these models are applied to populations that differ from the original training distributions. This consideration is particularly relevant for emotions such as fear and disgust, which may present greater sociocultural variability and are among the emotional categories most susceptible to automated misclassification.

Finally, future studies involving larger, more clinically stratified, and culturally diverse cohorts will be necessary to validate and generalize these preliminary observations. Longitudinal monitoring under naturalistic conditions may further clarify the stability and ecological validity of automated FER systems as potential digital biomarkers capable of identifying emotional vulnerability and relapse risk in individuals with substance use disorders. In addition, comparing FER models trained on datasets with different demographic and ethnic compositions, as well as developing locally annotated datasets containing spontaneous emotional expressions from culturally representative populations, may improve model adaptation, reduce algorithmic bias, and enhance the translational applicability of automated emotional monitoring systems in addiction rehabilitation settings.

## 5. Conclusions

The present study provides evidence that individuals with SUD exhibit altered emotional processing characterized by reduced responsiveness to positive stimuli, increased fear expression, and reduced disgust-related emotional responses. These findings support theoretical models that identify emotional dysregulation as a central mechanism underlying addiction. Furthermore, the results highlight the potential utility of computational facial emotion recognition technologies for the objective monitoring of emotional vulnerability during recovery.

## Figures and Tables

**Table 1:** Details of Participant Characteristics.

Variable	SUD		HC	
	$\%/\bar{x}$ ± SD	*n*	$\%/\bar{x}$ ± SD	*n*
Age	31.33 ± 10.01	22	25.79 ± 6.39	20
Education				
Secondary education	14.29%	3	0%	0
High School	47.62%	10	5%	1
Bachelor’s degree	23.81%	5	70%	14
Postgraduate degree	9.52%	2	25%	5
Employment				
Manual labor	42.86%	9	5%	1
Customer service	19.05%	4	5%	1
Self-employed	19.05%	4	10%	2
Healthcare	4.76%	1	30%	6
Unemployed	9.52%	2	5%	1
Other ^a^	4.76%	1	45%	9
Marital status				
Single	76.19%	16	85%	17
Long-term partner or married	4.76%	1	15%	3
Separation or divorce	19.05%	4	0%	0
Housing				
Alone	19.05%	4	5%	1
Parents and/or siblings	47.62%	10	60%	12
Partner with/without children	9.52%	2	15%	3
Relatives	19.05%	4	5%	1
Friends and/or roommates	4.76%	1	15%	3
Mental health				
Depressive disorders	52.38%	11	10%	2
Anxiety disorders	42.86%	9	20%	4
ADHD	28.57%	6	15%	3
Bipolar disorders	19.05%	4	-	-
Antisocial personality disorder	4.76%	1	-	-
None	19.05%	4	70%	14
Medications				
Antipsychotics	42.86%	9	-	-
Antidepressants	14.29%	3	-	-
Methylphenidate	9.52%	2	10%	2
Anxiolytics	9.52%	2	5%	1
Antiretrovirals	4.76%	1	-	-
Cardiovascular	4.76%	1	-	-
None	23.81%	5	85%	17

^a^ Financial support from family, scholarships and others.

**Table 2:** Mean values and group differences between HC and SUD across the observation period.

Variable	Group	Day (Mean ± SD)													
		1	2	3	4	5	6	7	8	9	10	11	12	13	14
Anxiety	SUD	2.67± 1.39	2.43± 1.33	2.28± 1.27	2.05± 1.20	2.19± 1.33	2.52± 1.21	2.71± 1.31	2.71± 1.55	2.19± 1.36	2.24± 1.37	2.5± 1.24	2.05± 1.16	2.24± 2.42	2.67± 1.49
	HC	1.56± 1.04	1.72± 1.07	1.28± 0.67	1.44± 0.78	1.44± 0.62	1.56± 0.78	1.33± 0.69	1.39± 0.78	1.67± 0.97	1.50± 0.62	1.39± 0.70	1.39± 0.50	1.70± 0.70	1.06± 0.42
	Difference *p* value	1.1 <0.01 *	0.71 0.08	1.0 <0.01 *	0.60 0.08	0.75 0.04 *	0.97 <0.01 *	1.4 <0.01 *	1.30 <0.01 *	0.52 0.18	0.74 0.04	0.66 0.05 *	0.66 0.03 *	0.79 0.04 *	1.6 <0.01 *
Somatic	SUD	0.47± 0.60	0.29± 0.46	0.43± 0.50	0.28± 0.46	0.43± 0.51	0.48± 0.51	0.43± 0.51	0.48± 0.51	0.43± 0.51	0.43± 0.51	0.48± 0.51	0.29± 0.46	0.33± 0.48	0.48± 0.51
	HC	0.33± 0.59	0.33± 0.48	0.33± 0.48	0.39± 0.50	0.17± 0.38	0.33± 0.48	0.22± 0.43	0.28± 0.46	0.22± 0.43	0.33± 0.48	0.44± 0.51	0.28± 0.46	0.28± 0.46	0.17± 0.38
	Difference *p* value	0.14 0.46	−0.04 0.76	0.09 0.55	−0.10 0.51	0.26 0.08	0.19 0.24	0.21 0.18	0.20 0.21	0.21 0.18	0.09 0.55	0.03 0.85	0.01 0.96	0.06 0.72	0.31 0.04
Preoccupation	SUD	0.52± 0.51	0.43± 0.51	0.33± 0.48	0.24± 0.44	0.38± 0.50	0.38± 0.50	0.38± 0.50	0.43± 0.51	0.38± 0.50	0.38± 0.50	0.43± 0.51	0.29± 0.46	0.33± 0.48	0.48± 0.51
	HC	0.39± 0.50	0.39± 0.50	0.22± 0.43	0.33± 0.49	0.28± 0.46	0.28± 0.46	0.28± 0.46	0.17± 0.38	0.39± 0.50	0.28± 0.46	0.56± 0.51	0.28± 0.46	0.22± 0.43	0.28± 0.46
	Difference *p* value	0.13 0.41	0.04 0.81	0.11 0.46	−0.09 0.52	0.10 0.51	0.10 0.51	0.10 0.51	0.26 0.08	−0.01 0.96	0.10 0.51	−0.13 0.44	0.01 0.96	0.15 0.46	0.20 0.21
Craving	SUD	0.48± 0.51	0.38± 0.50	0.48± 0.51	0.29± 0.46	0.24± 0.44	0.24± 0.44	0.38± 0.50	0.52± 0.51	0.33± 0.48	0.19± 0.40	0.19± 0.40	0.10± 0.40	0.29± 0.46	0.33± 0.48
	HC	0.00± 0.00	0.00± 0.00	0.00± 0.00	0.00± 0.00	0.00± 0.00	0.00± 0.00	0.00± 0.00	0.00± 0.00	0.00± 0.00	0.00± 0.00	0.00± 0.00	0.00± 0.00	0.00± 0.00	0.00± 0.00
	Difference *p* value	0.52 <0.01 *	0.38 <0.01 *	0.48 <0.01 *	0.29 0.01 *	0.24 0.03 *	0.24 0.03 *	0.38 <0.01 *	0.52 <0.01 *	0.33 <0.01 *	0.190 0.05	0.190 0.05	0.190 0.05	0.286 0.01 *	0.33 <0.01 *

* Items with a significative difference between groups.

## Data Availability

The datasets generated and analyzed during the current study are available from the corresponding author upon reasonable request and with authorization from the Centro Integral de Intervención Social, Fundación México Me Necesita A.C. The data are not publicly available due to ethical and privacy restrictions, as they contain sensitive clinical information that could potentially compromise the anonymity of the participant.

## Supplementary Materials

The following supporting information can be downloaded at: https://www.mdpi.com/article/10.3390/healthcare14101422/s1, Supplementary S1 (FER code), Supplementary S2 (Substance Use Characteristics of the SUD Group), Supplementary S3 (Comparison of valence and activation between groups by item).
