# Supplementary material for: Automated Facial Emotion Recognition System Detects Altered Emotional Processing During Craving Induction in Individuals with Substance Use Disorder
Source: Healthcare (Basel). 2026 May 21;14(10):1422. doi: 10.3390/healthcare14101422 (PMC13205282; doi:10.3390/healthcare14101422)
Supplement: Supplementary file 1 [file healthcare-14-01422-s001.zip › S1.pdf]

```
import cv2
import numpy as np
from tensorflow.keras.models import load_model
from tensorflow.keras.preprocessing.image import img_to_array

print("Import successful")

# Load the face detection model (Haar Cascade) and emotion model
face_cascade = cv2.CascadeClassifier(cv2.data.haarcascades +
'haarcascade_frontalface_default.xml')
emotion_model = load_model(r'C:/Users/bione/Desktop/Emociones/model_v6_23.hdf5')

# Emotion labels
emotions = ['Angry', 'Disgust', 'Fear', 'Happy', 'Sad', 'Surprise', 'Neutral']

# Initialize webcam
cap = cv2.VideoCapture(1)

# Initialize emotion counter
emotion_counts = {emotion: 0 for emotion in emotions}

while True:
  ret, frame = cap.read()
  if not ret:
    break

  gray = cv2.cvtColor(frame, cv2.COLOR_BGR2GRAY)
  faces = face_cascade.detectMultiScale(gray, scaleFactor=1.1, minNeighbors=5,
minSize=(30, 30))

  for (x, y, w, h) in faces:
    face_roi = gray[y:y+h, x:x+w]
    face_roi = cv2.resize(face_roi, (48, 48))
    face_roi = face_roi.astype("float") / 255.0
    face_roi = img_to_array(face_roi)

    preds = emotion_model.predict(np.expand_dims(face_roi, axis=0))[0]
    emotion_label = emotions[preds.argmax()]

    emotion_counts[emotion_label] += 1

    emotion_probabilities = ", ".join([f"{emotion}: {prob:.2f}" for emotion, prob in
zip(emotions, preds)])
```

```
        cv2.rectangle(frame, (x, y), (x + w, y + h), (0, 255, 0), 2)
        cv2.putText(frame, emotion_label, (x, y - 10), cv2.FONT_HERSHEY_SIMPLEX, 0.9, (0,
255, 0), 2)
        cv2.putText(frame, emotion_probabilities, (x, y + h + 20),
cv2.FONT_HERSHEY_SIMPLEX, 0.7, (0, 255, 0), 2)

    cv2.imshow("Emotion Detection", frame)

    if cv2.waitKey(1) & 0xFF == ord('q'):
        break

print("Emotion count summary:")
for emotion, count in emotion_counts.items():
    print(f"{emotion}: {count}")

cap.release()
cv2.destroyAllWindows()
```
