# Supplementary material for: Automated Facial Emotion Recognition System Detects Altered Emotional Processing During Craving Induction in Individuals with Substance Use Disorder
Source: Healthcare (Basel). 2026 May 21;14(10):1422. doi: 10.3390/healthcare14101422 (PMC13205282; doi:10.3390/healthcare14101422)
Supplement: Supplementary file 1 [file healthcare-14-01422-s001.zip › S2.pdf]

S2. Substance use characteristics of SUD group

Variable	% / $\bar{x}$ (SD)	*n*
**Highest impact**		
**Methamphetamine**	85.71%	18
**Alcohol**	9.52%	2
**Cocaine**	4.76%	1
**Years of use of highest impact substance**		
**Methamphetamine**	7.67 (5.93)	18
**Alcohol**	12.50 (16.26)	2
**Cocaine**	5	1
**Secondary substances**		
**Alcohol**	76.19%	16
**Tobacco**	66.67%	14
**Inhalants**	66.67%	14
**Cannabis**	61.90%	13
**Cocaine**	57.14%	12
**Hallucinogens**	23.81%	5
**Sedatives**	23.82%	5
**Other stimulants** [a]	23.82%	5
**Substance use onset**		
**Age**	14.81 (8.39)	22
**Min-Max**	6-48	
**Alcohol**	52.38%	11
**Tobacco**	47.62%	10
**Cannabis**	4.76%	1

[a] Other stimulants= designer psychoactive substances with unspecified composition.
