# Supplementary material for: Automated Facial Emotion Recognition System Detects Altered Emotional Processing During Craving Induction in Individuals with Substance Use Disorder
Source: Healthcare (Basel). 2026 May 21;14(10):1422. doi: 10.3390/healthcare14101422 (PMC13205282; doi:10.3390/healthcare14101422)
Supplement: Supplementary file 1 [file healthcare-14-01422-s001.zip › S3.pdf]

S3. Comparison of valence and activation between groups by item.

Item	Type	Category	Valence						Activation					
			**SUD**		**HC**		**Difference (Δ | *P* value)**		**SUD**		**HC**		**Difference**	
			Mean	±SD	Mean	±SD	(P value | Δ)		Mean	±SD	Mean	±SD	(P value | Δ)	
1	Positive	Animals / Pets	4.24	±0.89	4.35	±0.67	0.65|	-0.11	1.81	±0.68	2.10	±1.02	0.29|	-0.29
2	Positive	Food / Junk food	**2.71**	**±1.10**	**3.75**	**±0.91**	**<0.01|**	**-1.00***	**2.24**	**±0.70**	**2.85**	**±1.09**	**0.04|**	**-0.61***
3	Positive	People / Family	3.29	±1.10	3.90	±0.91	0.06|	-0.61	**2.05**	**±0.59**	**3.00**	**±1.12**	**<0.01|**	**-0.95***
4	Negative	Object / Blood	2.43	±0.87	2.55	±0.94	0.67|	-0.12	3.33	±0.97	3.70	±0.66	0.17|	-0.37
5	Substance	Alcohol	3.14	±0.96	3.15	±0.81	0.98|	-0.01	2.76	±0.77	2.80	±0.70	0.87|	-0.04
6	Neutral	Object / Home	2.95	±0.67	3.10	±0.55	0.45|	-0.15	2.90	±0.62	3.10	±0.55	0.30|	-0.20
7	Neutral	Object / Office	3.43	±0.75	3.35	±0.67	0.73|	0.08	2.62	±0.67	2.70	±0.73	0.71|	-0.08
8	Positive	Landscape / Water	3.95	±1.36	4.30	±0.86	0.34|	-0.35	1.52	±0.81	2.00	±1.17	0.14|	-0.48
9	Negative	Object / Medical	2.19	±1.12	1.85	±0.59	0.23|	0.34	3.62	±1.07	3.80	±0.70	0.53|	-0.18
10	Substance	Alcohol	2.76	±1.09	3.15	±0.81	0.21|	-0.39	2.81	±1.08	3.10	±0.55	0.29|	-0.29
11	Neutral	Object / Office	2.86	±0.73	3.00	±0.56	0.49|	-0.14	**2.62**	**±0.67**	**3.25**	**±0.55**	**<0.01|**	**-0.63***
12	Negative	Animals / Insect	2.48	±0.75	2.90	±0.91	0.11|	-0.42	**2.90**	**±0.89**	**3.55**	**±0.89**	**0.03|**	**-0.65***
13	Negative	People / Violence	**2.62**	**±1.02**	**3.35**	**±0.99**	**0.03|**	**-0.73***	3.10	±0.83	3.20	±0.95	0.71|	-0.10
14	Positive	Animals / Pets	4.05	±0.86	4.10	±0.97	0.86|	-0.05	1.81	±0.60	1.85	±0.99	0.87|	-0.04
15	Negative	People / Children	2.86	±0.79	2.70	±0.80	0.53|	0.16	3.29	±0.72	3.40	±0.60	0.58|	-0.11
16	Neutral	Object / Technology	**2.67**	**±0.66**	**3.15**	**±0.49**	**0.01|**	**-0.48***	2.71	±0.72	2.95	±0.51	0.23|	-0.24
17	Negative	Landscape / Death	2.67	±0.91	3.05	±0.89	0.18|	-0.38	3.14	±0.73	2.95	±0.83	0.43|	0.19
18	Positive	People / Fun	**2.76**	**±1.26**	**3.80**	**±1.06**	**<0.01|**	**-1.00***	**2.19**	**±0.87**	**3.10**	**±1.41**	**0.02|**	**-0.91***
19	Positive	Animals / Pets	3.86	±1.31	4.05	±0.94	0.59|	-0.19	1.90	±0.89	2.05	±1.10	0.64|	-0.15
20	Negative	Objects / Medical	2.48	±0.87	2.35	±0.67	0.61|	0.13	3.43	±0.93	3.60	±0.68	0.51|	-0.17
21	Positive	People / Sexual	**2.43**	**±0.98**	**3.70**	**±0.66**	**<0.01|**	**-1.30***	**2.24**	**±0.77**	**3.60**	**±0.68**	**<0.01|**	**-1.40***
22	Neutral	Object / Technology	3.00	±0.55	3.00	±0.00	>0.99|	0.00	**2.76**	**±0.54**	**3.15**	**±0.49**	**0.02|**	**-0.39***
23	Negative	Object / Medical	2.19	±0.81	2.20	±0.89	0.97|	-0.01	3.38	±1.16	3.95	±0.69	0.07|	-0.57
24	Negative	People / Violence	2.10	±0.94	2.10	±1.07	0.99|	0.00	3.76	±1.09	4.05	±0.69	0.32|	-0.29
25	Substance	Methamphetamine	2.48	±1.03	2.05	±0.69	0.13|	0.43	3.67	±0.91	3.45	±0.51	0.36|	0.22
26	Positive	People / Sports	**2.86**	**±0.91**	**3.90**	**±0.97**	**<0.01|**	**-1.00***	**2.19**	**±0.60**	**3.30**	**±0.86**	**<0.01|**	**-1.10***
27	Negative	Object / Death	2.81	±1.08	2.75	±0.72	0.84|	0.06	2.86	±0.91	3.25	±0.64	0.12|	-0.39
28	Substance	Cocaine	2.19	±1.12	2.10	±0.97	0.78|	0.09	3.62	±1.16	3.55	±0.69	0.82|	0.07
29	Neutral	Object / Home	3.33	±0.73	2.95	±0.51	0.06|	0.38	2.81	±0.60	2.90	±0.45	0.59|	-0.09
30	Positive	People / Children	2.95	±0.86	3.10	±0.85	0.59|	-0.15	**2.48**	**±0.75**	**3.20**	**±0.95**	**<0.01|**	**-0.72***
31	Negative	Object / Death	2.14	±0.91	2.25	±0.97	0.72|	-0.11	3.57	±0.98	3.80	±0.62	0.38|	-0.23

32	Positive	People / Sexual	**2.71**	**±1.01**	**3.65**	**±0.99**	**<0.01|**	**-0.94***	**2.33**	**±0.66**	**3.35**	**±0.93**	**<0.01|**	**-1.00***
33	Substance	Cigarette	2.33	±0.97	2.20	±0.89	0.65|	0.13	3.29	±1.01	3.55	±0.51	0.30|	-0.26
34	Substance	Pills	2.43	±0.93	2.25	±0.72	0.50|	0.18	3.10	±0.94	3.55	±0.76	0.10|	-0.45
35	Positive	Food / Junk food	3.00	±1.55	3.45	±1.28	0.32|	-0.45	**2.33**	**±1.20**	**3.25**	**±1.12**	**0.02|**	**-0.92***
36	Negative	Objects / Medical	2.81	±1.08	2.95	±0.60	0.61|	-0.14	3.05	±0.97	3.10	±0.45	0.83|	-0.05
37	Neutral	Objects / Job	3.33	±0.86	3.20	±0.52	0.55|	0.13	**2.67**	**±0.58**	**3.05**	**±0.39**	**0.02|**	**-0.38***
38	Substance	Marihuana	2.48	±1.12	2.10	±0.72	0.21|	0.38	3.19	±1.08	3.65	±0.59	0.10|	-0.46
39	Negative	People / Violence	1.95	±0.92	1.55	±0.76	0.14|	0.40	3.95	±0.97	3.80	±0.83	0.59|	0.15
40	Neutral	Landscape / Home	2.76	±1.04	2.80	±0.52	0.88|	-0.04	3.19	±1.08	2.95	±0.39	0.35|	0.24
41	Positive	People / Sports	**3.00**	**±1.30**	**3.95**	**±0.83**	**<0.01|**	**-0.95***	**2.14**	**±1.11**	**3.20**	**±1.01**	**<0.01|**	**-1.10***
42	Substance	Cigarette	2.33	±1.02	2.30	±0.73	0.91|	0.03	3.10	±0.94	3.50	±0.61	0.11|	-0.40
43	Neutral	Object / Home	**2.95**	**±0.50**	**2.90**	**±0.55**	**0.75|**	**0.05***	3.10	±0.54	3.05	±0.39	0.76|	0.05
44	Positive	People / Fun	3.43	±1.25	4.00	±0.92	0.10|	-0.57	**2.14**	**±1.11**	**2.70**	**±1.03**	**<0.01|**	**-0.56***
45	Negative	Object / Blood	2.19	±0.98	2.25	±0.97	0.85|	-0.06	3.43	±1.08	3.65	±0.49	0.41|	-0.22
46	Negative	People / Violence	2.10	±1.09	1.80	±0.83	0.34|	0.30	3.81	±1.12	3.95	±0.51	0.61|	-0.14
47	Neutral	Object / Home	2.76	±1.09	3.10	±0.64	0.24|	-0.34	2.76	±1.00	3.05	±0.60	0.27|	-0.29
48	Negative	People / Violence	1.90	±0.94	1.85	±0.99	0.86|	0.06	4.05	±0.92	4.05	±0.60	>0.99|	0.00
49	Positive	Landscape / Water	3.81	±1.21	4.50	±0.95	0.05|	-0.69	2.10	±1.14	2.00	±1.17	0.79|	0.10
50	Neutral	Food / Healthy food	2.86	±0.79	3.65	±0.75	<0.01|	-0.79	3.76	±0.77	2.60	±0.82	<0.01|	1.20

* Items with a significative difference between groups
